# Non-invasive detection of bone marrow fibrosis in myeloproliferative neoplasms using cell-free RNA

**DOI:** 10.1016/j.isci.2025.114325

**Published:** 2025-12-03

**Authors:** Mohamed Saad, Stijn N.R. Fuchs, Carmen Schalla, Katrin Götz, Jessica E. Pritchard, Niclas Flosdorf, Adam Benabid, Hélène F.E. Gleitz, Nils Leimkühler, Aurélien Dugourd, Rebekka K. Schneider

**Affiliations:** 1 Department of Cell and Tumor Biology, Faculty of Medicine, University Hospital RWTH Aachen, Aachen, Germany; 2 Department of Developmental Biology, Erasmus Medical Center, Rotterdam, the Netherlands; 3 Oncode Institute, Erasmus Medical Center Cancer Institute, Rotterdam, the Netherlands; 4 Institute for Computational Biomedicine, Bioquant, Faculty of Medicine, Heidelberg University and Heidelberg University Hospital, Heidelberg, Germany; 5 Department of Hematology and Stem Cell Transplantation, West-German Cancer Center, University Hospital Essen, Essen, Germany

**Keywords:** Molecular biology, Cancer

## Abstract

Myeloproliferative neoplasms (MPNs), particularly with myelofibrosis (MF), involve a disrupted perivascular hematopoietic niche, ultimately leading to bone marrow fibrosis. We asked if the transcriptome in cell-free RNA (cf-RNA) from the peripheral blood of patients with MPN (with JAK2V617F mutation) can detect bone marrow fibrosis. Transcriptomic profiling revealed significant gene expression changes correlating with reticulin fibrosis grades. Advanced reticulin fibrosis grades (2–3) showed upregulation of TGF-β pathways and extracellular matrix (ECM) remodeling markers, with decreased hematopoietic support. Grade 3 fibrosis was associated with increased proliferation signals and elevated inflammatory markers (S100A8/9). RUNX1 was identified as a key transcription factor in fibrosis, with its overexpression driving myofibroblast differentiation in mesenchymal stromal cells. IL-18 emerged as a critical inflammatory mediator, with elevated plasma levels correlating with the transformation to high-grade fibrosis (reticulin grades 2–3). Functional assays confirmed that the IL-18 stimulation of mesenchymal stromal cells induced fibrotic transformation, emphasizing its role as a biomarker and target.

## Highlights

• Peripheral cf-RNA profiling stratifies bone marrow fibrosis in patients with MPN
• RUNX1 drives stromal myofibroblast transformation
• IL-18 drives marrow fibrosis and marks transition to advanced disease stage

## Graphical abstract

**Figure:**
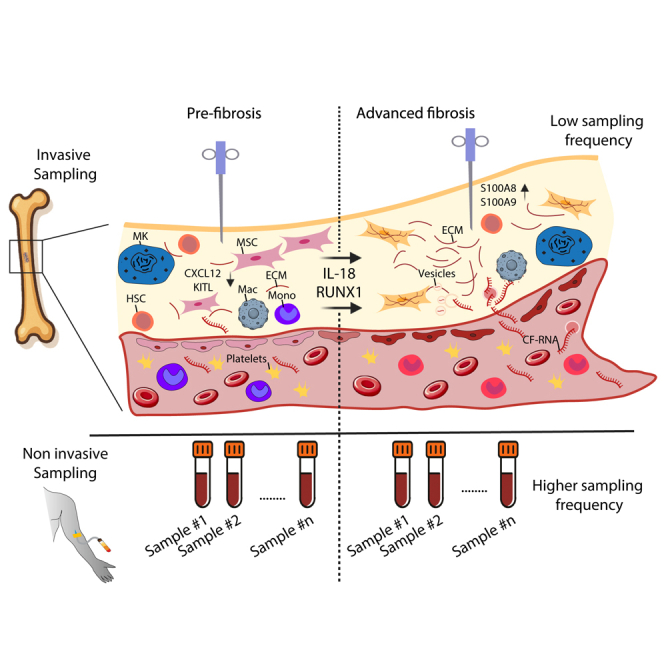

## Teaser

Myelofibrosis; Cell-free RNA; Inflammation; Hematopoiesis; Cancer

## Introduction

BCR::ABL1-negative myeloproliferative neoplasms (MPNs) arise from distinct mutations in hematopoietic stem cells (HSCs) that are mutually exclusive. The most common mutation is JAK2V617F, occurring in 60% of cases, followed by mutations in the *CALR* gene (20–25%) and the *MPL* gene (3–8%). MPNs, a type of chronic blood cancer, progress through two phases.^1^^,^^2^ Initially, the myeloproliferative phase is marked by an overproduction of mature blood cells. This phase eventually transitions to the myelofibrotic (MF) phase, characterized by bone marrow fibrosis that leads to the body’s inability to produce blood cells.

The assessment of bone marrow fibrosis in MPN classically relies on bone marrow biopsies. This invasive procedure hinders the continuous and serial monitoring of fibrosis progression from pre- (no reticulin fibrosis) to fibrosis onset (reticulin grade 1) and progression to advanced fibrosis (reticulin grades 2–3). Despite being generally safe, this invasive technique carries risks, including bleeding and infection. Additionally, bone marrow biopsies face methodological challenges, such as overlapping characteristics among essential thrombocythemia (ET), polycythemia vera (PV), and primary myelofibrosis (PMF), sampling errors due to non-homogeneous disease distribution in the bone, and limited insights into functional processes. These limitations underscore the urgent need for non-invasive diagnostic tools that can provide temporal insights into the fibrosis progression and support monitoring of fibrosis development in patients with MPN.

Peripheral blood, which circulates throughout the body and perfuses various tissues, including the bone marrow, offers a valuable resource for non-invasive diagnostics. The content of blood plasma includes cell-free nucleic acids, such as cell-free RNA (cf-RNA), which has shown promise in revealing pathological conditions in patients with cancer and physiological statuses during pregnancy.^2^^,^^3^ Cf-RNA is primarily derived from dead cells but can also be actively released through exosome shedding into the extracellular matrix.^4^^,^^5^

We hypothesize that a change in cf-RNA quality occurs in MPN with MF, and that is due to increased endothelial leakiness associated with the extensive remodeling of the perivascular niche during disease progression. Here, we focused on sequencing cf-RNA isolated from the plasma of peripheral blood in patients with MPN with different grades of fibrosis. We stratified the patients into four cohorts based on their corresponding bone marrow reticulin fibrosis grades. This approach allowed us to profile the transcriptomic alterations occurring during the stepwise transition from pre-fibrosis to overt fibrosis stages. Our results show that the transcriptome of cf-RNA can be used to stratify patients with MPN using a non-invasive technique and that this matches their reticulin grade. Additionally, our data reveals that cf-RNA from patients with high-grade fibrosis shows the same TGFβ signature as previously reported in the interaction of hyperplastic megakaryocytes in MPN with fibrosis-driving stromal cells.^6^ Finally, we identified an IL-18 driven inflammatory axis between the myeloid and stromal compartments as critical at the turning point from pre-fibrosis to overt fibrosis.

## Results

### The transcriptomic profile of circulating cell-free-RNA allows the stratification of patients with myeloproliferative neoplasms according to their fibrosis grade

**Figure 1:**
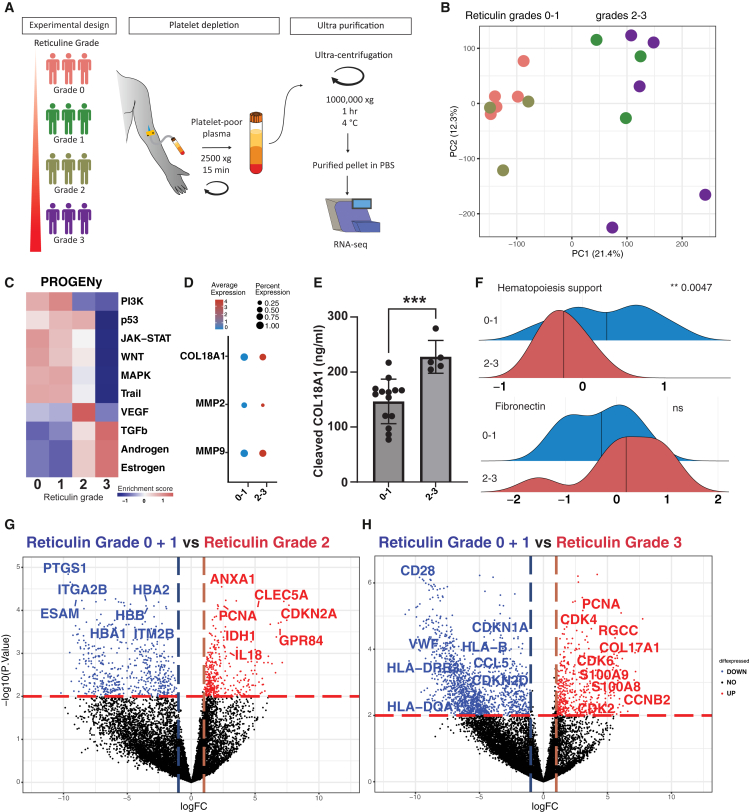
Stratification of patients with MF is possible based on their cf-RNA transcriptomic profiles (A) Overview of experimental design. Platelet-poor plasma samples were obtained from four patient groups with varying reticulin grades, followed by cf-RNA purification steps for RNA sequencing. (B) PCA of sequenced samples of patients with MF stratified according to reticulin grades. (C) PROGENy enrichment scores for each reticulin grade. (D) Dot plot compares the relative expression of extracellular matrix regulators MMP2/9 and COL1A1 between reticulin grades 0 and 1 and reticulin grades 2 and 3. Percent expression = number of samples expressing a particular gene/total number of samples in a cohort. (E) Bar plot shows cleaved COL18A1 (endostatin) protein level in plasma isolated from peripheral blood of patients diagnosed with pre-fibrosis (reticulin grades 0 and 1) and advanced fibrosis (reticulin grades 2 and 3). Bar plots are shown with mean ± SEM. (F) Ridge plot shows the enrichment score of a customized functional gene set, comparing mean expression levels in pre-fibrosis with advanced fibrosis. (G) Volcano plot depicts differentially expressed genes between combined pre-fibrosis (reticulin grades 0 and 1) and reticulin grade 2. H) Volcano plot shows differentially expressed genes between combined pre-fibrosis (reticulin grades 0 and 1) and reticulin grade 3. For (E, F), a *t* test was conducted using unpaired two-tailed settings. ∗∗∗*p* < 0.001, ∗∗*p* < 0.01, ns = 0.2070.

MPNs, particularly when associated with bone marrow fibrosis, are characterized by a disrupted hematopoietic niche and highly altered vasculature, leading to the deposition of fibrotic scar tissue.^7^^,^^8^ Peripheral blood reflects the MPN phenotype, and we hypothesized that changes in the extensively remodeled bone marrow niche would also be present in cf-RNA from peripheral blood. Our previous work has demonstrated significant changes in megakaryocyte-stromal cell cross-talk at the bone marrow perivascular niche.^6^ Therefore, we hypothesized that this would result in the release of cellular fragments and exosomes into the peripheral blood, which would reflect the pathognomonic features of MPN and MF in the bone marrow. We assembled a cohort of treatment patients with naive MPN (initial diagnosis) carrying the JAK2V617F driver mutation, representing all four reticulin fibrosis grades (Table S3). MF grading was performed by hematopathologists based on the WHO’s systematic bone marrow fibrosis grading criteria (Figure 1A).^9^ We focused on transcriptomic profiles obtained from cf-RNA by ultracentrifugation followed by bulk RNA sequencing to reflect a general overview of the intra- and extracellular dysregulation during different grades of fibrosis. After applying a quality control step to exclude genes with less than 1 CPM, 11,079 genes across all samples were identified. Principal component analysis (PCA) of the transcriptomic profiles showed a clear distinction between patients with pre-to low grade reticulin fibrosis (grades 0–1) and intermediate to advanced reticulin fibrosis (grades 2–3) (Figures 1B and S1A), reflecting 21.4% of the variance in gene expression for PC 1.

We aimed to understand the pathways responsible for differences in transcriptomic profiles between low grades (0–1) and advanced grades (2–3) reticulin fibrosis using PROGENy.^10^ The TGF-β pathway, a key regulator of bone marrow fibrosis, was upregulated in grades 2 and 3 but not in grades 0 and 1, indicating the effectiveness of cell-free transcriptomic analysis in reflecting bone marrow fibrotic niche dysregulation (Figure 1C). Additionally, higher expression of ECM regulators such as *MMP2/9* and *COL18A1* was found in grades 2–3 compared to grades 0–1, also highlighting that cf-RNA can reflect the ECM remodeling of the bone marrow (Figure 1D). At the protein level, we detected a corresponding significant upregulation in the cleaved fragment of COL18A1, Endostatin, in plasma from patients with advanced fibrosis compared to those diagnosed with profibrotic conditions (Figure 1E). In line with this, there was a trend that the fibronectin pathway was also upregulated in grades 2–3 compared to grades 0–1 (Figure 1F lower panel; Figure S1C).

We next conducted a pathway enrichment analysis based on established gene sets on hematopoiesis support of mesenchymal stromal subsets.^11^ Genes supporting normal hematopoiesis were significantly upregulated in reticulin grades 0–1 compared to grades 2–3, highlighting the advancement of the niche dysregulation in response to ECM remodeling (Figure 1F upper panel; Figure S1B).

To pinpoint the differences between reticulin grades 0–1 fibrosis to grades 2–3 at the gene level, we performed a differentially expressed gene (DEG) analysis. Grades 0 and 1 were combined, given their similarity as shown by PCA (Figure 1B). Two comparisons were performed: Low grade fibrosis (0 and 1) against grade 2, and low grade fibrosis (0 and 1) against grade 3. In grade 2, genes linked to cell proliferation, such as *CDKN2A* and *PCNA*, were upregulated compared to low grade fibrosis (146 differentially expressed genes) (Figure 1G). In grade 3 fibrosis, genes related to cell proliferation and cell cycle regulation were more upregulated compared to grade 0–1 fibrosis, in particular inflammation-related genes such as *S100A8/9* and fibrosis-associated genes (Figure 1H). Next, to benchmark cfRNA-derived signatures against cellular RNA, we compared our cfRNA data with published RNAseq profiles from CD34^+^ lineage (lin)^-^ hematopoietic stem and progenitor cells (HSPCs) obtained from peripheral blood of healthy mobilized donors and patients with myelofibrosis Figure S1D).^12^ This benchmarking analysis revealed a strong concordance between cfRNA and cellular RNA signatures, including the upregulation of pro-inflammatory mediators (IL18), extracellular matrix and fibrosis-associated genes (COL18A1, FN1, ACTA2), and proliferation markers such as PCNA. These results indicate that cfRNA captures key molecular features of myelofibrosis and faithfully reflects the fibrotic and inflammatory transcriptional programs observed in blood-derived cells, supporting its potential as a minimally invasive biomarker of disease activity.

### Increased cell cycle progression and decreased adaptive immunity are hallmarks of the progression of fibrosis

**Figure 2:**
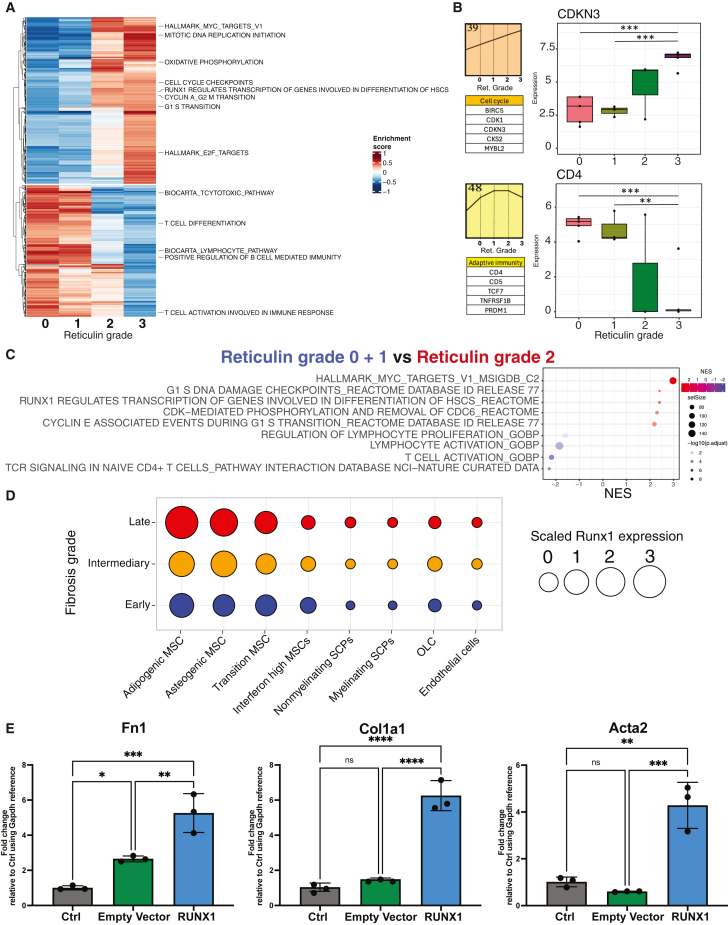
Progression from pre-to advanced fibrosis, coupled with cell cycle reprogramming and adaptive immunity dysregulation (A) Heatmap shows average normalized enrichment scores from GSVA pathway analysis using a collection of (Hallmark, Gene ontology, KEGG) databases, with hierarchical clustering across different fibrosis grades. (B) The top left panel displays the STEM pattern for the progression of proliferation signatures with increasing fibrosis grades. The lower left panel lists genes associated with the proliferation signature, and the right panel shows a boxplot of CDKN3 expression across different fibrosis grades. (C) The top left panel shows the STEM pattern for the consistent decline of the adaptive immunity signature with increasing fibrosis grades. The lower left panel lists genes associated with the adaptive immunity signature, and the right panel shows a boxplot of CD4 expression across different fibrosis grades. (D) Dot plot displays the GSEA normalized enrichment score for pairwise comparisons between the pre-fibrosis integrated cohort (grades 0 and 1) and grade 2. Schwann cell progenitors (SCPs) and osteoblastic lineage cells (OLC). (E and F) Dot plot shows scaled expression of Runx1 in stromal subpopulation isolated from a mouse model showing early, intermediate, and late stages of bone marrow fibrosis.11 (F) qRT-PCR analysis of Fn1, Col1a1, and Acta2 expression in bone marrow derived murine MSCs under normal conditions or upon transduction with either an empty vector or an overexpressing Runx1 vector. For panels (B–E), Bar plots and Boxplots are shown with mean ± SEM, one-way ANOVA with Tukey’s post-hoc test was used. ∗*p* < 0.05, ∗∗*p* < 0.01, ∗∗∗*p* < 0.001, ∗∗∗∗*p* < 0.0001.

To identify pathway dysregulation across samples, gene set variation analysis (GSVA)^13^ was used. The linear model from limma^14^ was applied to identify differentially enriched pathways between all fibrosis stages, using a threshold of FDR = 0.05 and log fold change = 0.5. GSVA enrichment was in line with DEGs analysis (Figures 1F and 1G) and confirmed that cell cycle regulation and proliferation signatures are upregulated in advanced fibrosis stages (grades 2–3) compared to reticulin grades 0–1 (Figure 2A). In advanced fibrosis, a downregulation of adaptive immune signals, particularly pathways related to CD4 positive T cell activation, was apparent (Figure 2A).

To further explore the gene expression patterns characterizing fibrosis progression, we applied the Short Time-series Expression Miner (STEM), a gene expression pattern recognition model.^15^ STEM analysis confirmed the increasing trend of cell proliferation and the decreasing trend of adaptive immunity throughout fibrosis progression (Figures 2B and 2C). STEM analysis highlighted a continuous increase in proliferation (pattern 39) throughout fibrosis progression and identified *CDKN3* as a key gene driving this pattern (Figure 2B, right panel).^16^ Conversely, STEM also showed a decrease in adaptive immunity (pattern 48) starting from grade 2 fibrosis (Figure 2C, left panel). CD4 was identified as a significant gene driving this trend (Figure 2C, right panel), in line with the previously observed decrease in CD4 positive T cells in advanced fibrosis.^17^

To focus on the transition from reticulin grades 0–1 to reticulin grades 2–3 of fibrosis, we applied GSEA^18^ to identify pathway alteration at this critical transition. In general, more pathways were significantly enriched in fibrosis grade 2 compared to grade 0–1, indicating a more intense dysregulation in the circulating transcriptomic profile in advanced fibrosis (Figure S2B). One of the top pathways identified in grade 2 fibrosis compared to grades 0–1 was RUNX1 regulating activity during HSC differentiation (Figure 2D).^19^ Using Cytoscape network analysis,^20^ we summarized the upregulated pathways from the GSEA pairwise comparison at the transition between reticulin grades 0–1 to reticulin grade 2 which confirmed the proliferation signal and RUNX1/3 pathway activation as one of the most significantly upregulated (Figure S2A).

To validate the connection between *RUNX1* expression and fibrosis in the dysregulated niche in MPN, we first asked the question of whether the JAK2V617F mutation leads to the increased expression of *Runx1*. HoxB8 cells transduced with the JAK2V617F mutation demonstrated a significant increase in *Runx1* expression compared to the cells expressing wild-type *JAK2* as a control, indicating that the increase in *Runx1* expression is driven by the MPN clone (Figures S2C and S2D). We next wondered if *Runx1* expression also influences the myofibroblast differentiation of mesenchymal stromal cells (MSCs), which we previously identified as the cellular driver of fibrosis.^6^^,^^11^ To address that, we analyzed our previously published single-cell RNA-seq dataset of mouse bone marrow stroma, which spans three fibrosis stages.^11^ We have noticed a gradual increase in *Runx1* expression from early to intermediate, which reaches its highest levels at the late fibrosis stage. Importantly, this upregulation is most pronounced in MSCs, the stromal subpopulations that we identified as the key drivers of fibrosis (Figure 2E). To validate this, we generated murine mesenchymal stromal cells (mMSCs) transduced with a *Runx1* overexpression plasmid significantly upregulated fibrosis-related genes such as *Fn1*, *Col1a1*, and *Acta2* compared to the empty vector (EV) control, suggesting a role for Runx1 in the dysregulated hematopoiesis in MPN but also showing a direct link to the fibrotic transformation (Figure 2F).

### Interleukin-18 is associated with the progression of bone marrow fibrosis in myeloproliferative neoplasms

**Figure 3:**
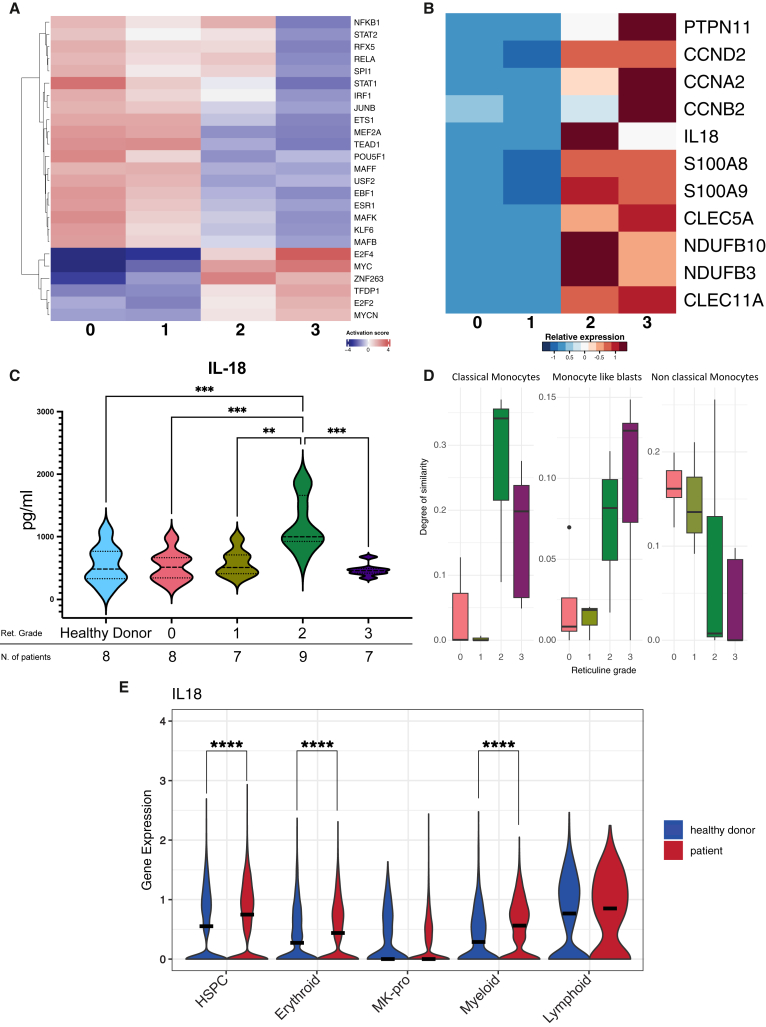
Analysis of gene expression profiles during the transition from early to advanced fibrosis reveals IL18 overexpression in the myeloid cell lineage (A) Dorothea inference analysis shows the scaled enrichment scores of the top 25 regulated transcription factors across different reticulin fibrosis grades. (B) Heatmap illustrates the expression pattern of NF-κB1 Dorothea-associated targets. (C) Violin plot depicts ELISA analysis results for IL-18 levels in human plasma of peripheral blood. (D) Boxplot from Cibersortx analysis shows the proportion of deconvolution of classical, non-classical, and blast-like monocytes within each fibrosis severity level. Boxplots are shown with mean ± SEM. (E) Violin plot illustrates IL18 expression pattern subclusters identified in PBMCs isolated from a patient with advanced MF.12 For (C), One-way ANOVA with Tukey’s post-hoc test was used. ∗*p* < 0.05, ∗∗*p* < 0.01, ∗∗∗*p* < 0.001. For (E), unpaired two-tailed t-tests were used. ∗*p* < 0.05, ∗∗*p* < 0.01, ∗∗∗*p* < 0.001, ∗∗∗∗*p* < 0.0001.

As we demonstrated a link between *Runx1* and the fibrotic transformation of fibrosis-driving cells, we next specifically interrogated the transcription factor (TF) activity by applying the DoRothEA model.^21^ TFs associated with cell cycle progression and proliferation as *MYC*, *E2F2*, and *E2F4*, were significantly upregulated in reticulin fibrosis grades 2 and 3 (Figure 3A) in line with the DEG and GSEA pairwise comparisons (compare Figures 1G, 1H, and 2A). Additionally, NF-κB1 was upregulated during the transition from grades 0–1 compared to grades 2–3 fibrosis with a distinct upregulation at grade 2 (Figure 3A). To identify the genes responsible for this pro-inflammatory expression pattern, we retrieved NF-κB1-related targets from the DoRothEA weighted database. Our findings highlighted a number of NF-κB1 targets known for their role in establishing an inflammatory environment in the bone marrow fibrotic niche (Figure 3B),^11^^,^^22^ among them *IL18*, *S100A8/S100A9*, *NDUFB10,* and *NDUFB3*. Specifically, the distinct *IL-18* expression peaking at grade 2 of reticulin fibrosis caught our attention (Figures 3B and 1F). IL-18 is recognized for its proinflammatory functions that contribute to fibrosis in various pathological conditions.^23^^,^^24^^,^^25^

To validate the altered transcriptomic profile of IL-18 on the protein level in MPN, we used an ELISA assay to measure IL-18 levels in the peripheral plasma of a healthy cohort and four cohorts of patients with MPN stratified by their fibrosis grade of MF (grades 0, 1, 2, 3). IL-18 levels significantly peaked in the plasma of patients with grade 2 reticulin (Figure 3C), in line with the transcriptome of the cf-RNA, further corroborating the importance of IL-18 during fibrosis progression. The association of clinical data with the IL-18 protein levels in patient plasma showed that high IL-18 levels correlated with a decrease in hemoglobin levels and an increase in leukocyte counts, most prominently at grade 2 reticulin fibrosis. A correlation with platelet counts was not observed (Figures S3A–S3C).

Thus, we next wondered if the increased expression of *IL18* in cf-RNA is derived from leukocytes. Using a scRNA-seq dataset from human bone marrow biopsy^26^ we built a reference matrix for CIBERSORTx,^27^ a machine learning model that allows for inferring cell-type-specific gene expression profiles without physical cell isolation. Our findings indicated an increased representation of special subclusters of monocytes in grade 2–3 reticulin fibrosis compared to grade 0–1 (Figure 3D). Based on this inference, classical monocytes were most prevalent in grade 2, corresponding with the IL-18 peak (Figure 3C). Monocytes and tissue-resident macrophages are well-described sources of IL-18 in various pathological conditions.^24^^,^^28^^,^^29^

We next investigated *IL18* expression in peripheral blood mononuclear cells (PBMCs) during homeostasis (healthy controls) and myelofibrosis using published single-cell transcriptomic datasets.^12^^,^^30^ In myelofibrosis PBMCs, *IL18* was significantly up-regulated in HSCPs, myeloid and erythroid cells (Figure 3E), confirming that increased *IL18* expression is associated with progressing fibrosis and that myeloid cells are a central source of this cytokine.

### Investigating the interleukin-18/interleukin-18R1 axis in human mesenchymal stromal cells

We next aimed to identify how IL-18 affects the fibrotic transformation in the bone marrow. Based on the data showing a significantly increased expression of IL-18 in myeloid and erythroid cells in patients with MPN diagnosed with advanced fibrosis, we hypothesized that IL-18 acts on stromal cell subsets expressing the IL-18 receptor 1 (IL18R1) in the bone marrow. Thus, we examined a human scRNA-sequencing dataset,^31^ which includes diverse fibroblast subclusters from various fibrosis affected tissues. *IL18R1* expression was detected in a subtype of fibroblasts characterized by high *ADAMDEC1* expression (Figure S4A–S4C).

**Figure 4:**
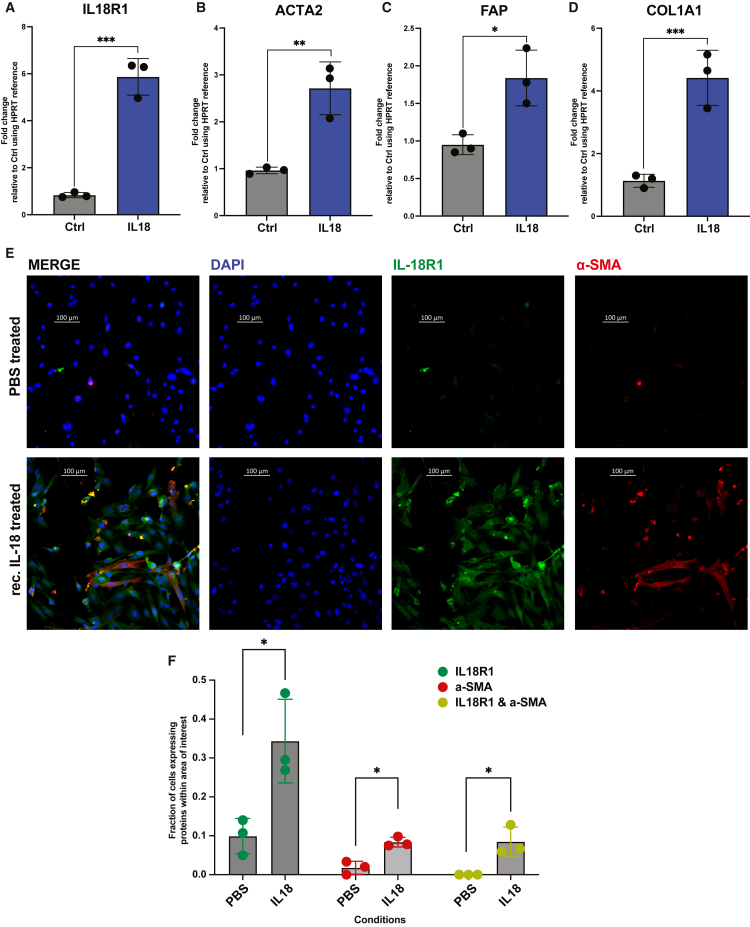
IL-18 pro-inflammatory stimulation induces fibrosis phenotype in bone marrow hMSCs (A–D) qRT-PCR analysis of IL18R1, COL1A1, FAP, and ɑ-SMA expression levels in hMSCs treated with either PBS or recombinant human IL-18 protein. (E) Immunofluorescence staining of hMSCs shows IL-18 expression in green and ɑ-SMA in red upon stimulation with 25 ng/mL IL-18 recombinant protein for 48 h. Representative images from three independent experiments are shown. (F) Bar plot depicts the normalized intensity quantification of IL-18R1 and ɑ-SMA, with overlapping cells represented in the plot. For panels (A–D) and (F), Bar plots are shown with mean ± SEM, unpaired two-tailed t-tests were used. ∗*p* < 0.05, ∗∗*p* < 0.01, ∗∗∗*p* < 0.001.

We next analyzed the response of bone marrow derived human mesenchymal stromal cells (hMSCs) to recombinant IL-18. The qRT-PCR confirmed an upregulation of *IL18R1* expression upon recombinant IL-18 exposure, confirming the stromal response to inflammatory cues (Figure 4A). IL-18 stimulation of hMSCs resulted in a significant increase in the expression of fibrosis-related genes such as *ACTA2*, *FAP*, and *COL1A1* (Figures 4B–4D), confirming the link between IL-18 and the fibrotic transformation of the bone marrow stroma. The increased expression of IL18R1 and aSMA upon IL-18 stimulation was further confirmed on the protein level by immunofluorescence, also highlighting a morphological switch to an activated MSC phenotype after IL-18 expression (Figures 3E and 3F).

## Discussion

Our characterization of circulating cf-RNA correlated with fibrosis grades of patients with MPN has established that 1) cf-RNA transcriptomes reflect changes in the bone marrow, 2) are a sustainable, non-invasive method to stratify patients with MPN in “low grade fibrosis” and “high grade fibrosis” with a turning point at grade 2 fibrosis and 3) identify regulating mechanisms in the fibrotic transformation in the bone marrow.

The enrichment of the TGF-β pathway in advanced fibrosis stages (grades 2 and 3) was a key finding to underline the validity of using cf-RNA to recapitulate the changes found in the fibrotic bone marrow niche in the peripheral blood. Previously, it has been demonstrated, even on the single cell level, that TGF-β is the main modulator of fibrosis and a characteristic of advanced fibrosis when up-regulated in MSCs as drivers of bone marrow fibrosis.^32^^,^^33^ Additionally, we observed higher expression levels of extracellular matrix regulators such as *MMP2/9* and *COL18A1* in advanced fibrosis compared to no-to-early fibrosis stages, corroborating their involvement in the remodeling of the bone marrow microenvironment.^34^ The higher expression of ECM peptides in patients with advanced fibrosis was also shown at the protein level of peripheral plasma using the cleaved form of COL18A1 as an example. This result highlights the capabilities of the ECM fragments isolated from peripheral plasma as a dynamic indicator of bone marrow fibrosis, as previously also highlighted by Hasselbalch and colleagues.^35^

RUNX1, a TF essential for hematopoiesis regulation, was significantly upregulated in advanced fibrosis and was a central hub regulating the proliferation of mutated hematopoietic cells and the fibrotic transformation of the stroma. We established a direct link between the expression of the JAK2V617F mutation and increased *Runx1* expression in mutated clones. Moreover, we demonstrated that the overexpression of *Runx1* in fibrosis-driving MSC induced their fibrotic transformation. This underscores the role of RUNX1 in mediating fibrotic transformation in the bone marrow, as previously documented in liver fibrosis.^36^

Our unbiased analysis of transcription factor activities revealed an upregulation of NF-κB activation, peaking at reticulin grade 2 during the transition from low grade to high grade bone marrow fibrosis. The transition was further marked by the enrichment of alarmins, indicating the establishment of an inflammatory, pro-fibrotic niche. This cf-RNA profile mirrors previous findings within the bone marrow niche, where NF-κB, the alarmins S100A8/S100A9, and TNFα prime the stromal niche toward inflammation and fibrosis.^6^^,^^11^^,^^37^ *IL18*, a downstream target of NF-κB, exhibited specific upregulation at the transition to more progressed fibrosis (reticulin grades 2–3), highlighting its significance in fibrosis progression. Monocytes and tissue-resident macrophages, identified as major sources of IL-18, were enriched in advanced fibrosis samples, aligning with the inflammatory microenvironment associated with fibrosis progression.^38^ Among the IL-1 family cytokines, IL-1β and IL-18, play pivotal roles in fibrosis progression.^23^^,^^39^ These cytokines share a common biosynthesis pathway initiated by the TLR4 receptor, which responds to pathogen-associated molecular patterns (PAMPs) and damage-associated molecular patterns (DAMPs).^40^ IL-18 has been recognized for its proinflammatory functions, significantly contributing to fibrosis progression in various pathological conditions.^23^^,^^41^^,^^42^ Our findings demonstrate that stimulation with recombinant IL-18 significantly upregulates IL-18R1 expression in hMSCs, confirming their responsiveness to inflammatory cues. Recombinant IL-18 further induced the differentiation of MSC into a-SMA positive myofibroblasts, establishing a direct link between IL-18 and fibrosis in the bone marrow. The plasma levels of IL-18 also indicate that IL-18 could mark the fibrosis progression in patients but bigger cohorts will be needed to establish the role of IL-18 as a potential biomarker.

These insights advance our understanding of inflammation-driven MF progression, highlighting the IL-18 inflammatory axis as a driver of inflammation and fibrosis. The roles of monocytes and macrophages in activating stromal cells within the IL-18 and IL-18R1 axis underscore the importance of the inflammatory microenvironment in fibrosis progression.

### Limitations of the study

Although the sample size across cohorts is relatively small, our benchmarking against peripheral blood RNAseq data supports the robustness of cfRNA-based signatures. These findings highlight the potential of cfRNA as a non-invasive biomarker for stratifying bone marrow fibrosis and monitoring disease activity, while emphasizing the need for validation in larger and more diverse patient populations, along with the inclusion of systematic comparison with whole blood or platelet RNA seq.

We also acknowledge that fibrosis should not be interpreted in isolation from other disease features. Nevertheless, multiple studies have shown that the degree of marrow fibrosis carries independent prognostic value across myeloproliferative neoplasms, with higher fibrosis grades associated with reduced survival and increased risk of disease progression. Therefore, identifying non-invasive cfRNA signatures predictive of marrow fibrosis is not only relevant for understanding disease biology but may also contribute to improved patient risk stratification and serve as a surrogate marker in therapeutic trials.

## Resource availability

### Lead contact

Further information and requests for resources and reagents should be directed to and will be fulfilled by the lead contact, Rebekka K. Schneider, MD, PhD (reschneider@ukaachen.de).

### Materials availability

This project did not include newly generated reagents.

### Data and code availability

• Normalized counts and metadata, along with differential gene expression results, were deposited in Zenodo https://doi.org/10.5281/zenodo.15496239. The fastq files can be retrieved from the GEO using the following ID: GSE308705 (https://www.ncbi.nlm.nih.gov/geo/query/acc.cgi?acc=GSE308705).
• This article does not include original code.
• Data shown in the article can be requested and will be fulfilled by the lead contact upon request for further analysis.

## Acknowledgments

R.K.S. is an Oncode Institute investigator and is supported by 10.13039/100008698ERC grants (Rewind-MF ERC-CoG 101124542; deFIBER ERC-StG 757339 and PoC DeAlarmin) and a ZonMW 10.13039/501100016061VIDI grant (09150172110021) and by 10.13039/501100001659Deutsche Forschungsgemeinschaft (DFG) (German Research Foundation; 504777725; 417911533; 514007497). The project also received funding from the program “10.13039/501100008848Netzwerke 2021,” an initiative of the Ministry of Culture and Science of the State of North Rhine Westphalia (CANTAR network). R.K.S. is the member of the E:MED Consortia Fibromap and the consortium CureFib, funded by the German Ministry of Education and Science (10.13039/501100002347BMBF). R.K.S is co-founder and shareholder of Sequantrix GmbH.

## Author contributions

M.S. performed experiments, conducted bioinformatics analysis, performed result interpretation, and wrote the article. S.N.R.F. collected and bio-archived patient data, performed experiments including isolation and sequencing of cell free RNA, analyzed data, and wrote and edited the article. N.B.L., H.F.E.G., and A.D. contributed to conceptualization, experimental design, data analysis, and interpretation, and edited the article.; C.S. and K. G. contributed to the knock-in experiments and IF staining. N.F. and A.B. generated the human cell lines, contributed to data analysis, and edited the article. R. K. S. designed and initiated the study, interpreted the data, received research funding, wrote the article, and organized the figures.

## Declaration of interests

The authors declare no competing interests directly related to this work. R.K.S., however, discloses unrelated funding, honorariums, and ownership as follows: R.K.S. has grants from Active Biotech. R.K.S. is the founder and shareholder of Sequantrix GmbH.

## STAR★Methods

### Key resources table

**Table:** 

REAGENT or RESOURCE	SOURCE	IDENTIFIER
**Antibodies**		

alpha-Smooth Muscle Actin Antibody	R&D	Cat #MAB1420; RRID: AB_262054
Interleukin-18 receptor 1	Sigma Aldrich	Cat #HPA007615; RRID: AB_1851675
goat anti-rabbit-Antibody	ThermoFisher	Cat #A-21245; RRID:AB_2535813
goat anti-mouse-Antibody	ThermoFisher	Cat #A-11005; RRID:AB_2534073
DAPI	Vector	Cat #H-1200-10; RRID:AB_2336790

**Bacterial and virus strains**		

pMIG_1xHA-huRUNX1	NA	NA

**Chemicals, peptides, and recombinant proteins**		

TRIZOL	Thermo Fisher	Cat #15596026
HyClone AlphaMEM	Cytiva	Cat #SH30265.01
Murine basic FGF	Peprotech	Cat #450-33
Murine EGF	Peprotech	Cat #315-09
Trypsin	Gibco	Cat #25300054

**Critical commercial assays**		

Human Total IL-18 DuoSet ELISA	R&D	Cat #DY318-05
Human Endostatin Duoset ELISA	R&D	Cat #DY1098
Fast SYBR Green Master Mix	Applied Biosystems	Cat #4385616
High-Capacity cDNA Reverse Transcription Kits	Thermo Fisher	Cat #4368814
Smart-Seq V4 Ultra-Low Input RNA Kit	Clontech Laboratories	Cat #634888
NucleoSpin RNA Plus XS kit	Macherey-Nagel	Cat #740990.50
High-Capacity cDNA Reverse Transcription Kit	Applied Biosystems	Cat #4368814
Fast SYBR Green Master Mix	Applied Biosystems	Cat #4385616

**Deposited data**		

Genome Reference Consortium Human Build 38 patch release 6, hg38	Genome Reference Consortium	https://www.ncbi.nlm.nih.gov/datasets/genome/GCF_000001405.32/
Raw Data	This paper	GSE308705
Normalized RNAseq counts	This paper	https://zenodo.org/records/15496239

**Oligonucleotides**		

See primer list in supplemental information	NA	NA

**Software and algorithms**		

R v4.3.2	CRAN	https://www.R-project.org
RStudio v3.6.3	Posit Software	https://posit.co/
TrimGalore v0.6.6	(Krueger et al., 2023)^43^	https://github.com/FelixKrueger/TrimGalore
STAR v2.7.9	(Dobin et al., 2013)^44^	https://github.com/alexdobin/STAR
Kallisto	(Bray et al., 2016)^45^	https://github.com/pachterlab/kallisto
HTSFilter v1.32.0	(Rau et al., 2013)^46^	https://github.com/andreamrau/HTSFilter
edgeR v3.42.4	(Robinson et al., 2010)^47^	https://bioconductor.org/packages/edgeR
limma v3.56.2	(Ritchie et al., 2015)^14^	https://bioconductor.org/packages/limma/
Progeny v1.16.0	(Schubert et al., 2018)^10^	https://github.com/saezlab/progeny
DoRothEA v1.6.0	(Garcia-Alonso et al., 2019)^21^	https://github.com/saezlab/dorothea
clusterProfiler v4.8.3	(Wu et., 2021)^48^	https://bioconductor.org/packages/clusterProfiler/
Cytoscape v3.7	(Paul et al., 2003)^20^	https://cytoscape.org/
Cibersortx	(Newmann et al., 2019)^27^	https://cibersortx.stanford.edu/
STEM v1.3.13	(Ernst et al., 2006)^15^	https://www.cs.cmu.edu/∼jernst/stem/
GraphPadPrism v8	GraphPad Software Inc	RRID:SCR_002798
Qupath v0.5.0	NA	https://qupath.github.io/

### Experimental model and study participant details

Peripheral blood samples (*N* = 16 patients) were collected with consent from the Hematology, Oncology, Hemostaseology and Stem Cell Transplantation departments of RWTH Aachen University with an informed written consent upon approval from the Ethikkommission an der Medizinischen Fakultät der Rheinisch-Technischen Hochschule (RWTH) Aachen (EK127/12, EK206/09 and EK099/14 respectively). Moreover, some of the peripheral blood samples were also collected from Erasmus Medisch Centrum Rotterdam upon having a consent form from patients upon having an ethical vote MEC-2018-1445 and from patients with MPN following an ethical vote MEC-2018-1504 by the Medisch Ethische Toetsings Commissie (METC), Rotterdam. Clinical metadata are listed in Tables S3 and S4. Bone marrow biopsies were fixed, embedded in paraffin, sectioned, and graded according to WHO fibrosis standards at RWTH University Hospital.^9^

### Method details

#### Sample processing and RNA library preparation

Peripheral blood samples were collected using EDTA as an anticoagulant. The samples were centrifuged at 1500 xg for 15 min, and the supernatant was subsequently collected. This supernatant underwent a second centrifugation at 2500 xg for 15 min to obtain Platelet-Poor Plasma (PPP). A volume of 350 μL PPP was mixed with 28 mL of phosphate-buffered saline (PBS) and then ultracentrifuged at 100000 xg for 1 h at 4°C using a Beckman Optima XPN-100. The resulting pellet, containing extracellular content, was dissolved in 100 μL of PBS and then combined with 800 μL of TRIZOL LS Reagent (ThermoFisher). RNA was extracted through a phenol-chloroform method and cDNA libraries were prepared using the Smart-Seq V4 ultra-low input RNA kit from Clontech Laboratories, following the manufacturer’s instructions. The amplified cDNA was further processed to create Illumina-compatible libraries according to the TruSeq Nano DNA sample preparation guide (Illumina). These libraries were then subjected to paired-end sequencing (2x75 cycles) on an Illumina HiSeq2500 platform.

#### ELISA

IL-18: Peripheral blood samples were collected with EDTA, centrifuged at 1500 xg for 15 min to obtain plasma, and stored at −80°C. For the ELISA assay, samples were thawed and centrifuged at 2500 xg for 5 min. Plasma was diluted 1:10, and IL-18 levels were measured using the Human Total IL-18 DuoSet ELISA kit provided by R&D. The assay involved reconstituting standards, preparing samples, coating plates with capture antibody overnight, and adding a detection antibody. Absorbance at 450 nm and 540 nm was read with the CLARIOstar Plus. IL-18 concentrations were quantified using a four-parameter logistic fit in GraphPad Prism Software.

Cleaved COL18A1 (Endostatin): Peripheral blood samples were collected in EDTA tubes, centrifuged at 2500 xg for 7 min to obtain plasma, and stored at −20°C. For the ELISA assay, samples were thawed at 30°C and centrifuged at 2500 xg for 7 min. Plasma was diluted 1:300 in 1% BSA (05470-5G, Sigma-Aldrich) in PBS, and endostatin levels were measured using the Human Endostatin DuoSet ELISA kit (DY1098) provided by R&D systems. The assay was performed in accordance with the manufacturer datasheet. Absorbance at 450 nm and 560 nm was measured with the Victor X3 plate reader. Endostatin concentrations were quantified using a four-parameter logistic fit in GraphPad Prism Software.

#### Immortalized murine bone marrow stromal cells

Murine primary stromal cells were isolated and cultured as described previously.^49^ Once the cells reached 70–80% confluence, they were immortalized by overexpressing hTERT and large T vectors. The cells were maintained in AlphaMEM (CytiviaHyClone) with 20% FCS, 1 ng/mL mouse Fibroblast Growth Factor (FGF), 5 ng/mL mouse EGF, both provided by peprotech, and 1% Pen/Strep provided by Gibco. After 5–7 passages, the cells were transduced with either a Runx1 overexpression retrovirus or an empty PMIG control retrovirus, followed by GFP-positive selection. The Runx1 vector was prepared as previously detailed.^50^ Quality control check was implemented to control for mycoplasma contamination.

#### Immortalized human bone marrow stromal cells

Human bone marrow stromal cells derived from femoral head was established and maintained as described previously.^51^ Quality control check was implemented to control for mycoplasma contamination.

#### RNA isolation and qRT-PCR

RNA was extracted using the NucleoSpin RNA kit (Macherey-Nagel) according to the manufacturer’s protocol. cDNA synthesis was performed with the High-Capacity cDNA Reverse Transcription Kit (Applied Biosystems) and Murine RNase Inhibitor (New England Biolabs). The cDNA synthesis included cycles at 25°C for 10 min, 37°C for 120 min, 85°C for 5 s, and then held at 4°C. The cDNA was stored at −20°C. Gene expression was analyzed using the StepOnePlus Real-Time PCR system (Applied Biosystems) with SYBR Green fluorescence (Applied Biosystems). qRT-PCR was carried out following the program outlined in Table S2. Mouse Gapdh was used for normalizing mouse gene expression, while human HPRT was used for human genes (see Table S3 for primer details).

#### Immunocytochemistry

For immunofluorescence analysis of human immortalized MSCs, cells were incubated for 48 h with either PBS or 25 ng/mL human recombinant IL-18. After incubation, cells were stained overnight at 4°C with primary antibodies against IL-18R1 (1:100, HPA007615), from Abcam and α-SMA (1:100, MAB1420) from R&D. Secondary staining was done with AF-647 goat anti-rabbit (1:500, #A-21245) for IL-18R1 from Thermo Fisher Scientific and AF-648 goat anti-mouse (1:500, #A-11005) for α-SMA from from Thermo Fisher Scientific, along with DAPI (Vector Laboratories, H-1200-10). Fluorescence intensity was measured using Zeiss Zen Pro (version 3.2) and analyzed semi-quantitatively with Qupath (version 0.5.0).

### Quantification and statistical analysis

#### Statistical analysis

Statistical analyses were conducted using GraphPad Prism (version 9.0.0). An unpaired *t* test and multiple t-tests were used for comparing two groups, while a two-way ANOVA with Tukey’s multiple comparisons test was used for more than two groups. Customized statistical models were applied for bioinformatics analyses of bulk and scRNA-seq data, as described in the RNA analysis section.

#### Bulk RNA-Seq analysis

The sequencing data was initially trimmed using TrimGalore (version 0.6.6), followed by alignment with STAR software (version 2.7.9).^44^ Gencode (version 38)^52^ was employed as the reference for gene annotation. Read quantification was conducted using Kallisto^45^ to generate a raw count matrix. Quality control included filtering out lowly expressed genes with HTSFilter (version 1.32.0).^46^ Counts per million (CPM) normalization from the edgeR package (version 3.42.4)^47^ was then applied to prepare the matrix for Principal Component Analysis (PCA) and differential gene expression analysis using limma (version 3.56.2).^14^ Limma analysis was conducted with default parameters to calculate the corrected false discovery rate (FDR) for all contrasts across different fibrosis grades. All RNA analysis was performed using R version 4.3.2 and RStudio 3.6.3.

#### PROGENy pathway inference analysis

For network-based pathway enrichment analysis, we used Pathway RespOnsive GENes for activity inference (PROGENy) (version 1.16.0),^10^ leveraging the human database. Our raw count matrix was filtered to include differentially expressed genes from six key comparisons between fibrosis grades (grade_1 vs. grade_0, grade_2 vs. grade_1, grade_3 vs. grade_2, grade_2 vs. grade_0, and grade_3 vs. grade_0) with *p*-values ≤0.01 and log2fc ≥ 2. This filtered count matrix was log2 CPM normalized and served as the input for the PROGENy analysis. Additionally, we filtered the PROGENy module matrix to include only pathways with more than 180 targets.

#### Dorothea transcription factor interaction enrichment

Transcription factor interactions were inferred using DoRothEA (version 1.6.0).^21^ The human database from DoRothEA was used, and default settings were applied to generate normalized enrichment scores for transcription factors across all fibrosis grades, based on the developer recommendations. Confidence levels A, B, and C were specified, and the parameter `eset_filter = FALSE` was added within the VIPER application.

#### GSEA/Cytoscape

Gene Set Enrichment Analysis (GSEA) was performed using various gene set databases from the Molecular Signatures Database (MSigDB), including hallmark, KEGG, GO, and REACTOME datasets. The integrated MSigDB was downloaded from [BaderLab] (https://download.baderlab.org/EM_Genesets/July_01_2020/Human/symbol/Human_GOBP_AllPathways_no_GO_iea_July_01_2020_symbol.gmt). The GSEA function from the clusterProfiler package (version 4.8.3) in R was run with default parameters.^48^ This analysis compared pre-fibrosis grades (grades 0 and 1 combined) against grades 2 and 3.

For Cytoscape network analysis, GSEA was conducted specifically for the grade_2 vs. Grade_1 comparison using GSEA software (version 4.3.0) with a pre-ranked gene list (-log10(gene_an FDR) x log2fc sign), derived from limma pairwise analysis. The results were then analyzed with the Enrichment Map application (version 3.3.6) in Cytoscape (version 3.7).^20^

#### Cibersortx

Cibersortx analysis was conducted with consistent normalization for both query and reference datasets, as recommended.^27^ For organ deconvolution, Transcripts Per Million (TPM) normalization was used to align with the TPM-normalized reference organ count matrix from the Human Protein Atlas (version 20).^53^ Cellular contribution inference used CPM normalization for the reference matrix,^26^ matching our CPM-normalized query count matrix. Signature matrices were created using the Cibersortx Create Signature Matrix option, with settings: Disable quantile normalization = True, Minimum value of expression = 0.1, Number of replicates = 3, Kappa = 999, q value = 0.01, Filtration of non-hematopoietic genes = False, Number of barcode genes = 3,000–5,000.

#### Customized gene set enrichment

We created a gene set for the hematopoiesis support signature as described previously,^11^ and used the fibronectin signature from Reactome^54^ to aggregate gene expression. A ridgeplot was used to visualize and display the distribution of genes in each signature. Samples from grade 0 and grade 1 were combined into a pre-fibrosis cohort, while grade 2 and grade 3 samples formed the advanced fibrosis cohort.

#### STEM

Gene expression patterns were analyzed using STEM (version 1.3.13).^15^ The log2 CPM count matrix from all fibrosis grades was filtered to exclude non-differentially expressed genes. STEM was configured with log2 CPM normalization and a maximum profile change of 2 units. A permutation test used point 0 as the starting point, with human EBI and GO terms for pathway progression. GO terms were filtered to 5–500 genes per term, requiring at least 3 significant terms per cluster, and multiple hypothesis correction was applied.

#### Analysis of published scRNA-seq datasets

We analyzed previously published scRNA-seq datasets from bone marrow aspirates,^30^ peripheral blood mononuclear cells (PBMC),^12^ and fibroblasts^31^ using Seurat (version 4.0). Initial preprocessing, quality control, and cell annotation were performed as recommended by the authors. Our analysis focused on the post-normalized data, particularly the expression levels of IL18 and IL18R1 in the previously annotated cell types across different tissues.
