# Non-invasive detection of bone marrow fibrosis in myeloproliferative neoplasms using cell-free RNA

**DOI:** 10.1016/j.isci.2026.114899

**Published:** 2026-02-05

**Authors:** Mohamed Saad, Stijn N.R. Fuchs, Carmen Schalla, Katrin Götz, Jessica E. Pritchard, Niclas Flosdorf, Adam Benabid, Hélène F.E. Gleitz, Nils Leimkühler, Aurélien Dugourd, Rebekka K. Schneider

## Main text

(iScience *29*, 114325; January 16, 2026)

In the originally published version of this article, two errors were identified in Figure 1. Specifically, in Figure 1A, the colors for reticuline group grades 1 and 2 were swapped in the depiction of the experimental design, and the centrifugal speed for ultra-purification was mentioned as 1000,000 xg instead of 100,000 xg. This error has now been corrected in the article online. The authors apologize for the inconvenience and assure that these changes do not alter the main findings and conclusions of the manuscript.

**Figure:**
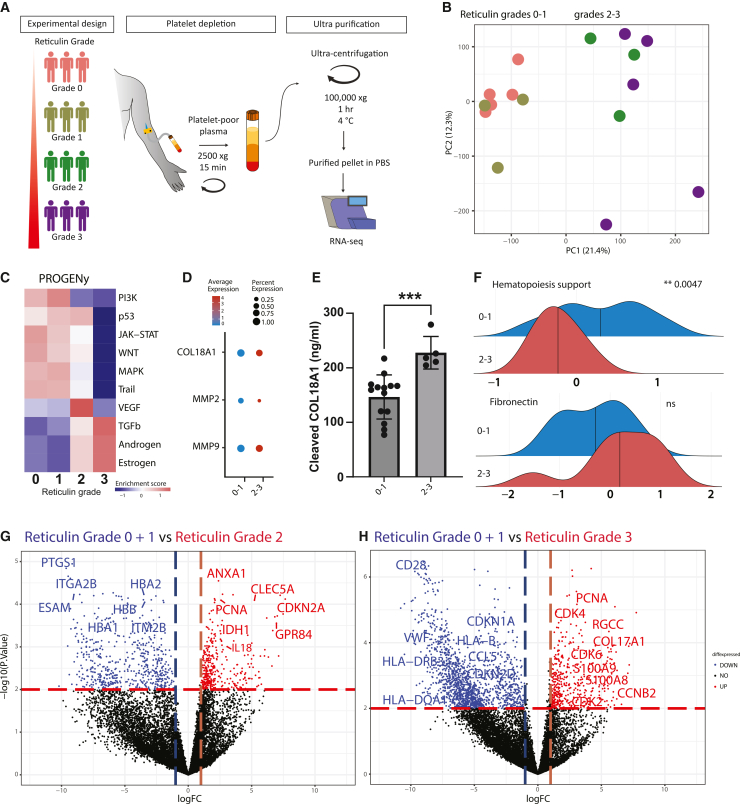
Figure 1. Stratification of patients with MF is possible based on their cf-RNA transcriptomic profiles (corrected)

**Figure:**
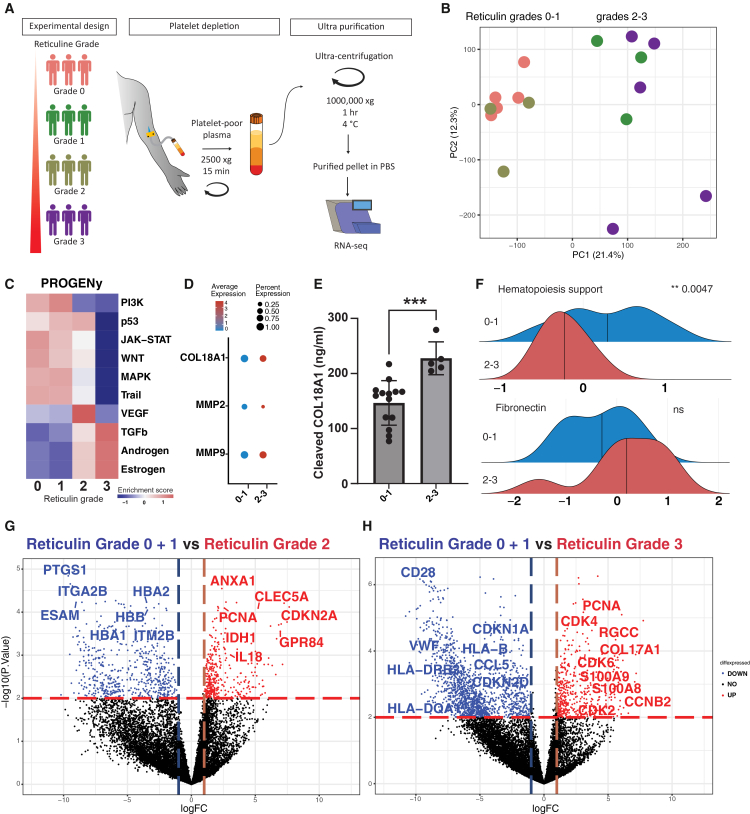
Figure 1. Stratification of patients with MF is possible based on their cf-RNA transcriptomic profiles (original)

