# Supplementary material for: Non-invasive detection of bone marrow fibrosis in myeloproliferative neoplasms using cell-free RNA
Source: iScience. 2025 Dec 3;29(1):114325. doi: 10.1016/j.isci.2025.114325 (PMC12834115; doi:10.1016/j.isci.2025.114325)
Supplement: Document S1. Figures S1–S5 and Tables S1–S6 [file mmc1.pdf]

iScience, Volume *29*

Supplemental information

Non-invasive detection of bone marrow fibrosis in myeloproliferative neoplasms using cell-free RNA

Mohamed Saad, Stijn N.R. Fuchs, Carmen Schalla, Katrin Götz, Jessica E. Pritchard, Niclas Flosdorf, Adam Benabid, Hélène F.E. Gleitz, Nils Leimkühler, Aurélien Dugourd, and Rebekka K. Schneider

Non-Invasive Detection of Bone Marrow Fibrosis in Myeloproliferative Neoplasms Using Cell-Free RNA

Mohamed Saad, [1#] Stijn N.R. Fuchs, [2,3#] Carmen Schalla, [1] Katrin Götz, [1] Jessica E. Pritchard, [1,2,3] Niclas Flosdorf, [1] Adam Benabid, [1] Hélène F.E. Gleitz, [2,3] Nils Leimkühler, [5] Aurélien Dugourd, [4] Rebekka K. Schneider [1,2,3*]

1 Department of Cell and Tumor Biology, Faculty of Medicine, University Hospital RWTH Aachen, Aachen, Germany

2 Department of Developmental Biology, Erasmus Medical Center, Rotterdam, the Netherlands

3 Oncode Institute, Erasmus Medical Center Cancer Institute, Rotterdam, the Netherlands

4 Institute for Computational Biomedicine, Bioquant, Faculty of Medicine, Heidelberg University and Heidelberg University Hospital, Heidelberg, Germany

5 Department of Hematology and Stem Cell Transplantation, West-German Cancer Center, University Hospital Essen, Essen, Germany

# These authors contributed equally

***Lead author**

Rebekka K. Schneider

Department of Cell and Tumor Biology

University Hospital RWTH Aachen

Email: reschneider@ukaachen.de

Phone: +49 241 8038540

Supplementary figures:

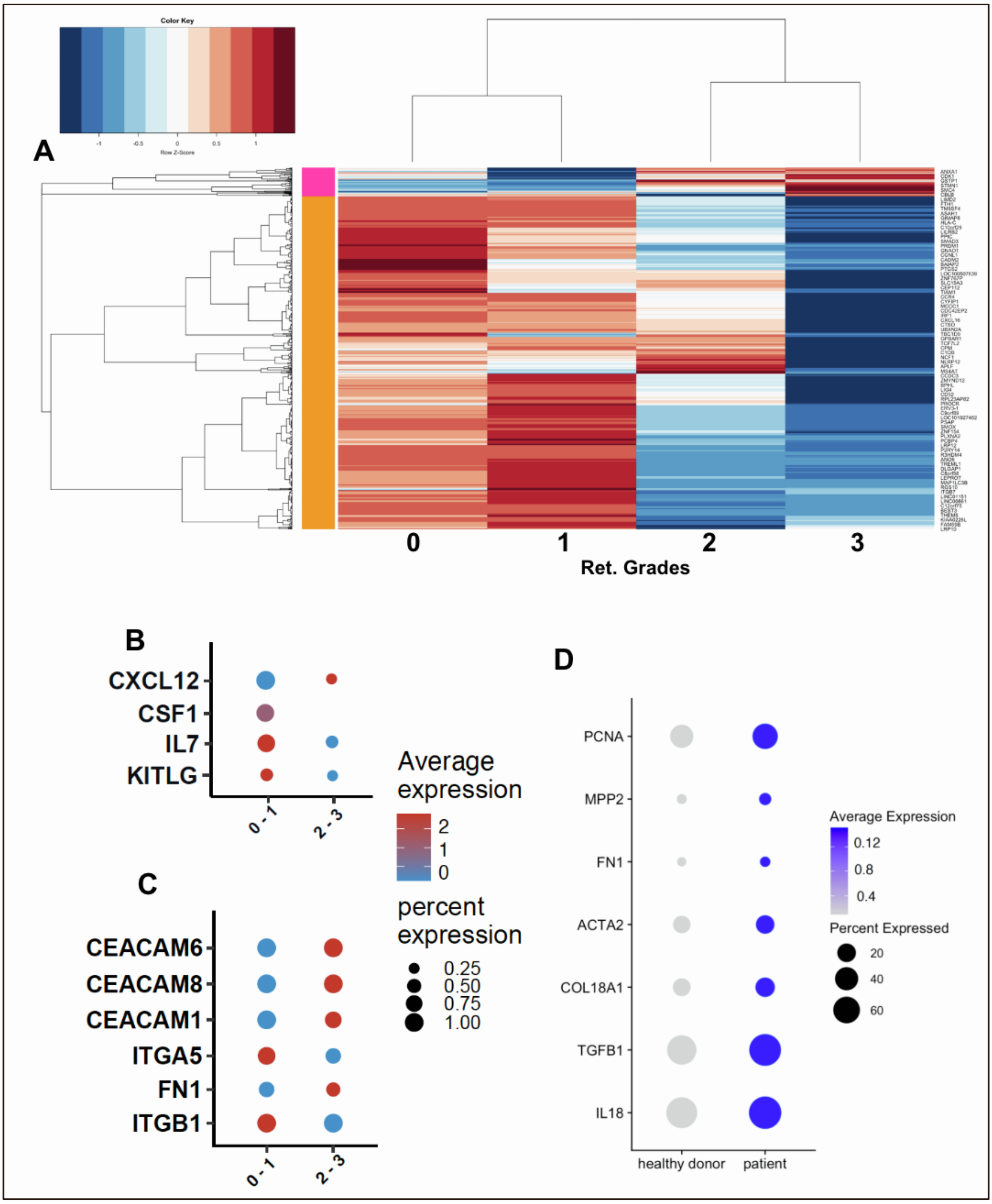

Supplementary Figure 1. In-depth illustration of marker gene patterns between early and advanced stages of fibrosis.

A) Hierarchical clustering heatmap of fibrosis grades, based on gene expression modules, showing 779 differentially expressed genes (p-value = 0.05, logfc = 2) from DEG analyses, including comparisons of Grade_1vs0, Grade_2vs0, Grade_3vs0, Grade_2vs1, Grade_3vs1, and Grade_3vs2.

B) and C) Dot plots depicting the relative expression of all genes in the customized gene set enrichment related to hematopoiesis support and fibronectin-related genes, as shown in Figure 1E. Percent expression = number of samples expressing a particular gene / total number of samples in a cohort.

D) Normalized expression level of selected markers in CD34+ lineage (lin)− HSPC compartment isolated from peripheral blood samples from healthy mobilized apheresis donors and patients with myelofibrosis (Psaila et al. 2020).(1)

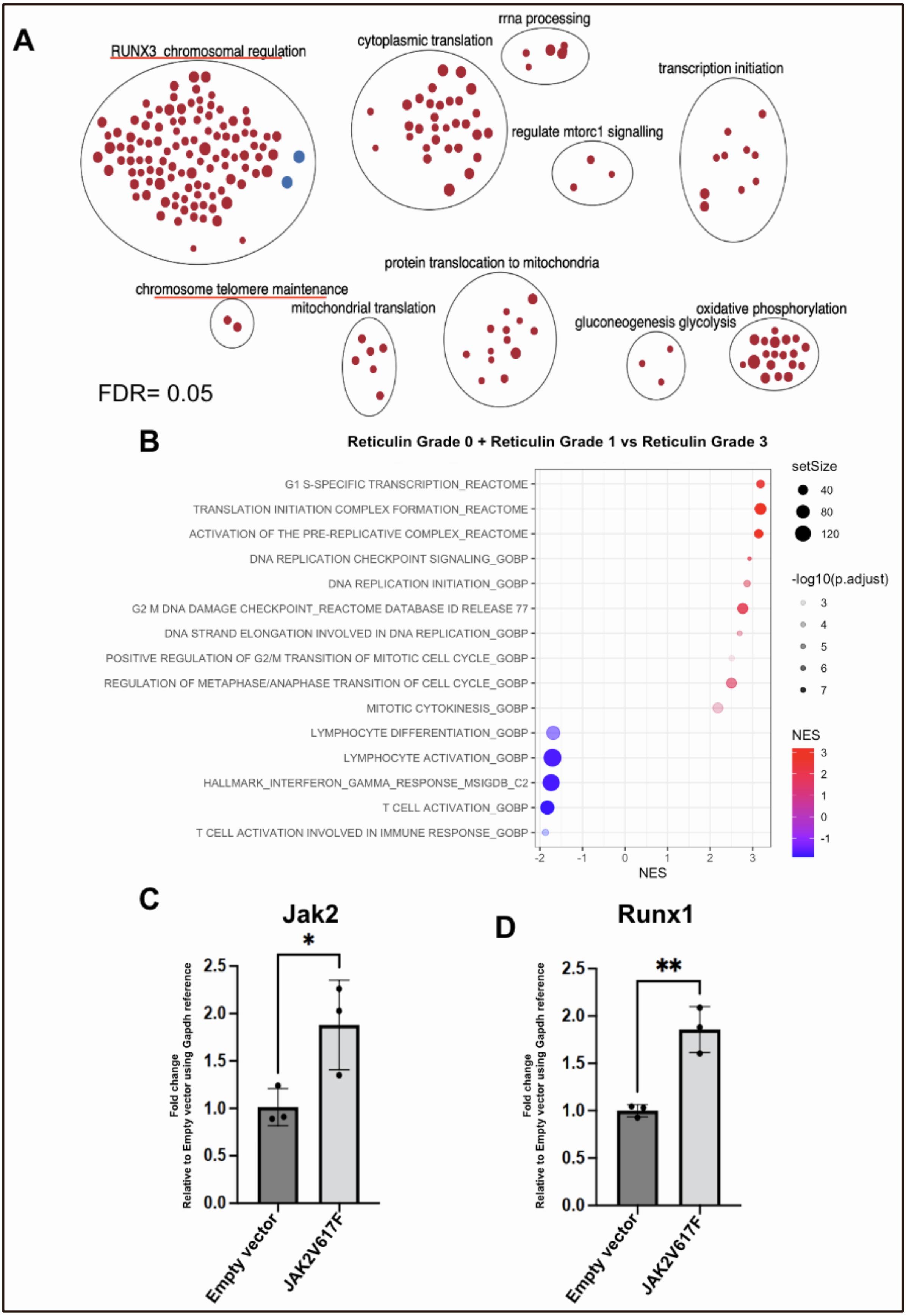
A
RUNX3_chromosomal regulation
cytoplasmic translation
rrna processing
transcription initiation
regulate mtorc1 signalling
chromosome telomere maintenance
mitochondrial translation
protein translocation to mitochondria
gluconeogenesis glycolysis
oxidative phosphorylation
FDR= 0.05
B
Reticulin Grade 0 + Reticulin Grade 1 vs Reticulin Grade 3
G1 S-SPECIFIC TRANSCRIPTION_REACTOME
TRANSLATION INITIATION COMPLEX FORMATION_REACTOME
ACTIVATION OF THE PRE-REPLICATIVE COMPLEX_REACTOME
DNA REPLICATION CHECKPOINT SIGNALING_GOBP
DNA REPLICATION INITIATION_GOBP
G2 M DNA DAMAGE CHECKPOINT_REACTOME DATABASE ID RELEASE 77
DNA STRAND ELONGATION INVOLVED IN DNA REPLICATION_GOBP
POSITIVE REGULATION OF G2/M TRANSITION OF MITOTIC CELL CYCLE_GOBP
REGULATION OF METAPHASE/ANAPHASE TRANSITION OF CELL CYCLE_GOBP
MITOTIC CYTOKINESIS_GOBP
LYMPHOCYTE DIFFERENTIATION_GOBP
LYMPHOCYTE ACTIVATION_GOBP
HALLMARK_INTERFERON_GAMMA_RESPONSE_MSIGDB_C2
T CELL ACTIVATION_GOBP
T CELL ACTIVATION INVOLVED IN IMMUNE RESPONSE_GOBP
-2 -1 0 1 2 3
NES
setSize
40
80
120
-log10(p.adjust)
3
4
5
6
7
NES
3
2
1
0
-1
C
Jak2
*
Fold change
Relative to Empty vector using Gapdh reference
2.5
2.0
1.5
1.0
0.5
0.0
Empty vector
JAK2V617F
D
Runx1
**
Fold change
Relative to Empty vector using Gapdh reference
2.5
2.0
1.5
1.0
0.5
0.0
Empty vector
JAK2V617F

Supplementary Figure 2. RUNX1 drives the enrichment of the proliferation signature at the transition point from pre-fibrosis to advanced fibrosis stages.

A) Cytoscape network analysis illustrating the differentially regulated pathways identified through GSEA by comparing pre-fibrosis grades to advanced fibrosis grade 2. B) Dot plot showing the GSEA normalized enrichment score for the pairwise comparison between the integrated pre-fibrosis cohort (Grades 0 and 1) and Grade 3. C) qRT-PCR analysis of Jak2 and Runx1 in HOxB8 cells derived from murine bone marrow and transduced with either an empty vector or the JAK2V617F mutation.

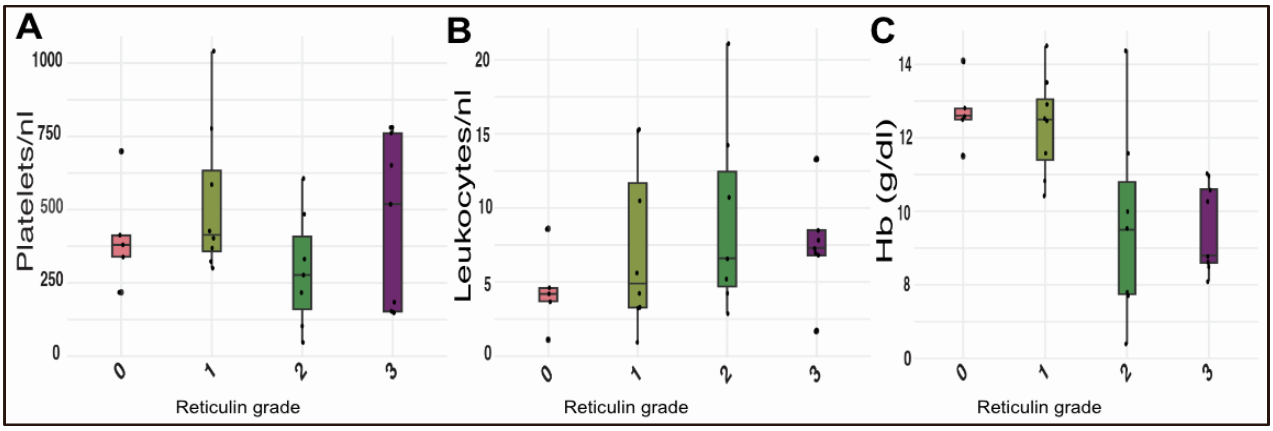

Supplementary Figure 3. Clinical profiling of MF patients integrated into the ELISA analysis.

A-C) Box plots depicting hemoglobin, leukocyte, and platelet levels in patients included in the ELISA analysis.

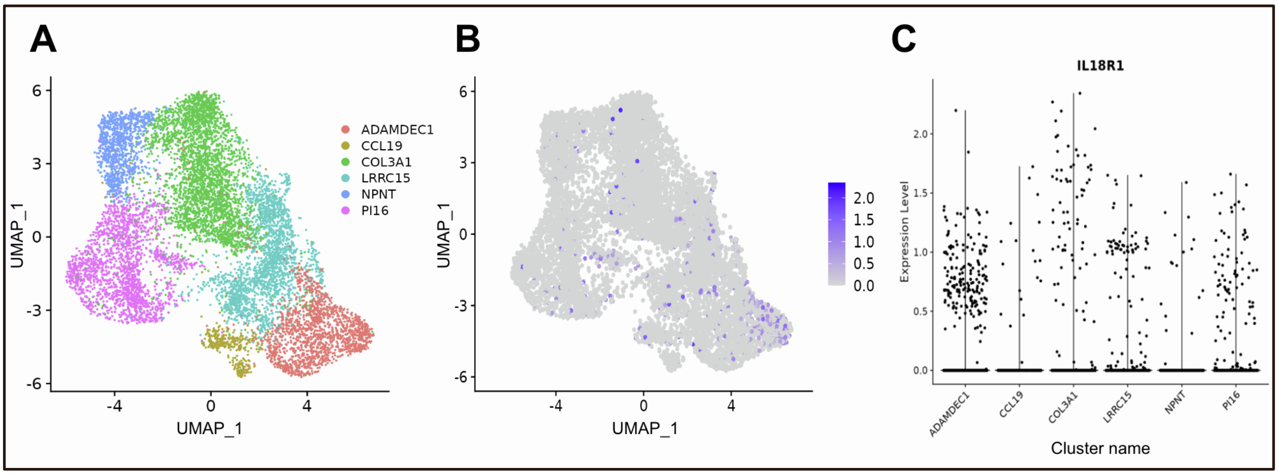

Supplementary Figure 4. Transcriptomic data from human bone marrow suggest that stromal cells receive IL-18 produced by HSPCs and monocytes.

A-C) Source dataset from (2). A) UMAP visualization of cell types. B) UMAP visualization of IL18R1 expression. C) Violin plot for IL18R1 expression.

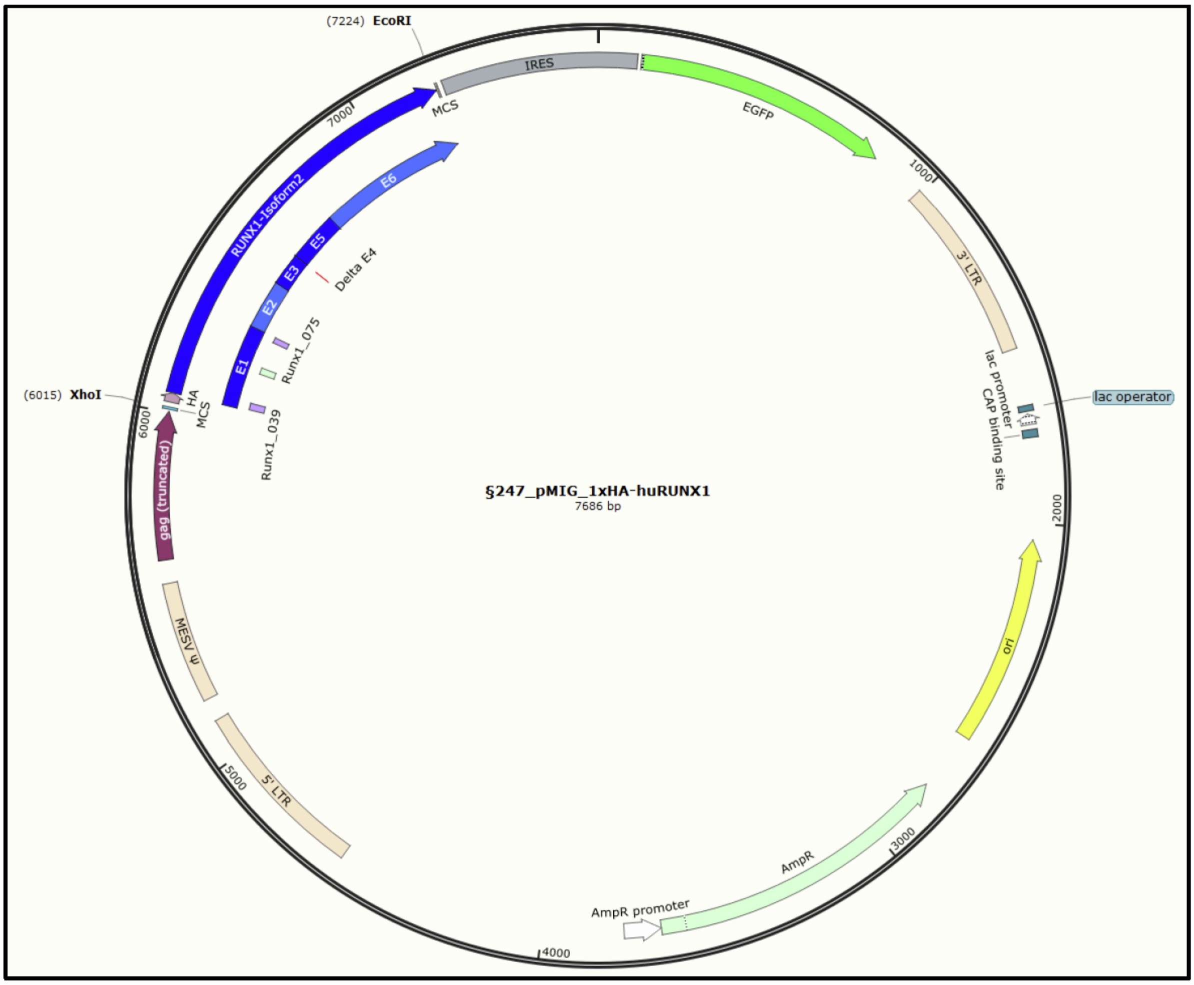

Supplementary Figure 5. pMIG_1xHA-huRUNX1 Vector Map

A schematic illustrating the RUNX1 vector utilized for RUNX1 overexpression.

Supplementary tables:

Supplementary Table 1. Reagents for Reverse Transcriptase Reactions

Reagents required for the reverse transcriptase process to convert mRNA into cDNA.

Reagent (Stock concentration)	Volume per 1 sample	Company
10× RT Buffer, 1.0 mL (ThermoFisher: 4368814)	2µl	Thermo Fisher Scientific
10× RT Random Primers, 1.0 mL (ThermoFisher: 4368814)	2µl	Thermo Fisher Scientific
25X dNTP Mix (100 mM) (ThermoFisher: 4368814)	0.5µl	Thermo Fisher Scientific
MultiScribeTM Reverse Transcriptase, 50 U/µL (ThermoFisher: 4368814)	0.8µl	Thermo Fisher Scientific
RNase Inhibitor murine (NEB)	1µl	NEB
RNA (1 µg)	13.7µl	NA

Supplementary Table 2. qRT-PCR Procedure Steps

qRT-PCR cycles and corresponding incubation times.

Temperature (°C)	Time (min:sec	Cycles (n)
95	0:20	1x
95	0:03	1x
60	0:30	40x
95<br>60 + 0.3 to 95<br>95	00:15<br>00:60<br>00:15	1x<br>1x<br>1x
4	infinity	1x

Supplementary Table 3. Clinical Metadata for RNA-seq Patient Cohorts

Information on MF grade as determined by the pathology department at RWTH UKAachen Hospital, along with the MPN pathological category for each patient on the day of plasma collection. Each patient was assigned an experimental ID to maintain anonymity.

Patient_Experimental_ID	Disease	Age	Fibrosis grade	Treatment	Mutation	Gender	Ancestry
Patient_1	ET	71	0	No treatment	JAK2V617F	Female	European
Patient_2	PV	60	0	No treatment	JAK2V617F	Female	European
Patient_3	PV	59	0	No treatment	JAK2V617F	Male	European
Patient_4	PV	60	0	No treatment	JAK2V617F	Male	European
Patient_5	PV	63	0	No treatment	JAK2V617F	Male	European
Patient_6	ET-MF	75	1	No treatment	JAK2V617F	Male	European
Patient_7	PMF	53	1	No treatment	JAK2V617F	Female	European
Patient_8	MPN-U vs. ET	26	1	No treatment	JAK2V617F	Male	European
Patient_9	Post-PV-MF	54	2	No treatment	JAK2V617F	Female	European
Patient_10	PMF	63	2	No treatment	JAK2V617F	Male	European
Patient_11	Post-ET-MF	72	2	No treatment	JAK2V617F	Male	European
Patient_12	PMF	65	3	No treatment	JAK2V617F	Female	European
Patient_13	PMF	71	3	No treatment	JAK2V617F	Male	European
Patient_14	PMF	42	3	No treatment	JAK2V617F	Male	European
Patient_15	PMF	66	3	No treatment	JAK2V617F	Male	European
Patient_16	PMF	62	3	No treatment	JAK2V617F	Male	European

Supplementary Table 4. Clinical Metadata for ELISA Experiment Patients

Details of the MPN pathological subtype for each patient according to clinical department diagnoses, along with the bone marrow fibrotic grade from the pathology department at RWTH UK Aachen. Included are normalized ELISA measurements and standard results from routine blood counts.

Patient_ ID	Disease	Bone marrow Reticulin grade	Conc_IL-18 pg/ml	Leukocytes/nl	platelets/nl	Hb (g/dl)	Gender	Ancestry
Patient_1	ET	0	673.74607	3.6	218	13.5	Female	European
Patient_2	PV	0	975.747743	6.2	699	15.1	Male	European
Patient_3	PV	0	231.520542	7.1	339	13.8	Male	European
Patient_4	PV	0	390.295324	11.1	380	12.5	Female	European
Patient_5	PV	0	637.466392	6.7	412	13.6	Female	European
Patient_6	PV	0	632.770249	7	354	14.5	Female	European
Patient_7	PV	0	323.881538	5	280	13.2	Male	European
Patient_8	PV	0	388.109203	4.6	258	12.5	Male	European
patient_9	PV	1	408.231761	5.7	324	11.4	Female	European
patient_10	PV	1	506.643	5.8	369	13.9	Female	European
patient_11	PV	1	671.181314	6.7	300	13.5	Female	European
patient_12	PV	1	962.105014	17.7	1040	11.8	Male	European
patient_13	PV	1	708.001184	17.8	402	12.6	Male	European
patient_14	PV	1	354.805534	3.4	426	13.5	Female	European
patient_15	ET	1	444.441289	8.1	585	15.5	Male	European
patient_16	PV	2	891.493026	16.7	484	15.4	Male	European
patient_17	PV	2	1855.63708	7.7	277	8.7	Female	European
patient_18	Post-PV-MF	2	982.932049	9.1	218	10.5	Male	European
patient_19	PMF	2	1056.13506	23.6	46	7.4	Female	European
patient_20	Post ET-MF	2	998.799095	13.2	102	12.6	Male	European
patient_21	PMF	2	655.382969	6.7	607	11	Female	European
patient_22	PMF	2	1866.48926	5.4	332	8.8	Male	European
patient_23	PMF	2	955.693399	5.4	466	11	Female	European
patient_24	PMF	2	1465.27316	9.8	152	9.5	Female	European
patient_25	PMF	3	490.108794	4.2	149	9.6	Female	European
patient_26	Post ET-MF	3	340.19204	11	519	9.1	Female	European
patient_27	PMF	3	416.370876	10.3	652	11.6	Female	European
patient_28	PMF	3	473.362958	9.5	183	11.3	Female	European

patient_29	Post-ET-MF	3	456.697397	9.8	152	9.5	Female	European
patient_30	PMF	3	675.472829	9.3	761	9.8	Female	European
patient_31	PMF	3	418.769702	15.8	780	12	Female	European

Supplementary Table 5. List of Primers for qRT-PCR Analysis in Human and Mouse cDNA

primers used for qRT-PCR measurements from human and mouse cDNA samples.

Primer name	Sequence (5′ – 3′)
GAPDH_F_human	GAAGATGGTGATGGGATTTC
GAPDH_R_human	AGGGGAGATGTTCTGGGAAC
IL18R1_F_human	AAGAACGCGCAGGTTTGAGAT
IL18R1_R_human	GAGCAGTTGAGCCTTACGTTT
α-SMA_F_human	TCC TTC ATC GGG ATG GAG TCT
α-SMA_R_human	TAC ATAGT GGTG CCC CCT GA
FAP_F_human	ACC CAC GCT CTG AAG CAG
FAP_R_human	ACG ATT TTT ACC AAG TTC TCA TTT
COL1A1_F_human	ATC AAG GTC GCT GTC CTC CGT
COL1A1_R_human	CAC CGG AGG GCG AGG TTC TT
Col1a1_F_Mouse	ACG GCT GCA CGA GTC ACA C
Col1a1_R_Mouse	GGC AGG CGG GAG GTC TTT
Fn1_F_Mouse	ATC TGG ACC CTC TCC TGA TAG T
Fn1_R_Mouse	GCC CAG TGA TTT CAG CAA AGG
Jak2_F_Mouse	GAT TTC AGG CCT GTC TTT CAG
Jak2_R_Mouse	ATG TTC CTT GTT GCC AGG TC
Runx1_F_Mouse	CTC CGT GTC TAC CAC TCA CT
Runx1_R_Mouse	ATG ACG GTG ACC AGA GTG C
α-Sma_F_Mouse	CAT CTC CAG AGT CCA GCA CA
α-Sma_R_Mouse	CAT CTC CAG AGT CCA GCA CA
GAPDH_F_Mouse	AGG TCG GTG TGA ACG GAT TTG
GAPDH_R_Mouse	TGT AGC ACC ATG TAG TTG AGG TCA

Supplementary Table 6. Reagents for Serum-Free Medium and Cell Culture

A list of serum-free medium and other cell culture reagents utilized in the study.

Component	Supplier (Catalog number)	Stock concentration	Final concentration
IMDM	Thermo Fisher Scientific (12-440-061)	100%	50%
DMEM	Capricorn Scientific (DMEM-HA)	100%	50%
RPMI 1640 medium	Gibco-Life Technologies (31870-074)	100%	50%
Phosphate buffered saline (PBS)	Gibco-Life Technologies (31870-074)	100%	100%
Fetal Bovine serum (FBS)	Gibco-Life Technologies (31870-074)	100%	100%
Trypsin-EDTA	Gibco-Life Technologies (31870-074)	100%	100%
Red blood cells lysis buffer	BD Bioscience	100%	10%
DMSO	Serva	100%	100%

References:

1. Psaila B, Wang G, Rodriguez-Meira A, Li R, Heuston EF, Murphy L, et al. Single-Cell Analyses Reveal Megakaryocyte-Biased Hematopoiesis in Myelofibrosis and Identify Mutant Clone-Specific Targets. Mol Cell. 2020 May;78(3):477-492.e8.

2. Buechler MB, Pradhan RN, Krishnamurty AT, Cox C, Calviello AK, Wang AW, et al. Cross-tissue organization of the fibroblast lineage. Nature. 2021 May 27;593(7860):575–9.
